# Predictors and Moderators of Spontaneous Pretend Play in Children with and without Autism Spectrum Disorder

**DOI:** 10.3389/fpsyg.2016.01577

**Published:** 2016-10-13

**Authors:** Erin Kang, Eliana F. Klein, Angeline S. Lillard, Matthew D. Lerner

**Affiliations:** ^1^Department of Psychology, Stony Brook University, Stony BrookNY, USA; ^2^Department of Psychology, University of Virginia, CharlottesvilleVA, USA

**Keywords:** autism spectrum disorder, spontaneous pretend play, assessment, cognitive development, observational coding

## Abstract

Although pretend play has long been linked to children’s normative cognitive development, inconsistent findings call for greater rigor in examining this relation (Lillard et al., 2013). Spontaneous pretend play is often impacted in atypical development, notably in autism spectrum disorder (ASD). Since ASD traits exist along a continuum in the general population, investigating how pretend play varies across the range of ASD symptoms by indexing variations in ASD traits in both typically developing and ASD populations may provide insight into how ASD symptoms may influence the relation between pretend play and associated processes in cognitive development. This study used rigorous observational methods to assess spontaneous pretend play. Specifically, 5-min free-play sessions with two discrete toy sets were double-coded by blinded coders (coder assignment counterbalanced). Key facets of pretense development [attribution of pretend properties (APP), object substitution (OS), imaginary objects] were examined. These facets of pretend play production were then analyzed in relation to ASD symptoms, as well as plausible, long-theorized correlates [theory of mind (ToM), verbal ability, familiarity, and interest in specific toys]. Forty children (*M*_age_ = 6;5, *SD*_age_ = 1.45; 29 males), six of whom met the threshold for ASD diagnosis via parent-reported ASD symptoms, participated in play sessions and completed measures of verbal IQ and ToM. Besides the measure of child ASD symptoms, parents completed a survey of their child’s interest in and familiarity with the play session toys. Overall, greater ToM predicted more APP, and more interest in the toys presented predicted more OS. In terms of overall pretend play production, two results were counterintuitive. First, among children with more ASD symptoms, verbal ability marginally negatively predicted pretend play production. Second, among children with fewer ASD symptoms, ToM negatively predicted pretend play production. Further probing revealed that the negative effect of ASD symptoms on pretend play was simultaneously moderated by both variables: low ToM and high verbal ability both related to less pretend play production among children with more ASD symptoms. Implications for assessment and subsequent treatment for pretend ability among children with varying degrees of ASD symptoms, as well as for future research, are discussed.

## Introduction

Play is a common feature of childhood. While exploring the world around them, children begin to play with everyday objects (e.g., pots and pans), more typical objects (e.g., dolls and toy cars), and eventually with their peers. By the time they are 3 years old, most typically developing (TD) children independently and spontaneously engage in symbolic or pretend play, such as pretending a banana is a telephone (Lillard, 2015). Pretend play is a combination of play and pretense, or the “stretching (of) one reality over another” (Lillard, 1993). The ability to pretend requires a metarepresentational ability, or the ability to hold onto two mental representations in the mind (Leslie, 1987; Lillard, 1993). The first reflects the state of the real world, or the perceived situation (e.g., here is a cup, the cup is empty), and the second reflects the pretend situation (e.g., this cup that is really empty contains tea; Leslie, 1987) that the pretender needs to be able to map onto the contexts of the real world.

Although, increasingly sophisticated pretend naturally emerges in TD children in their second year of life (Haight and Miller, 1993), the development of pretend appears impaired for autism spectrum disorder (ASD) children, even for those who are “high-functioning” with intact verbal ability (Jarrold, 2003). Even though children with ASD might pretend less and differently, research consistently suggests that many children with ASD *do* still pretend (Baron-Cohen, 1987; Libby et al., 1998; Rutherford and Rogers, 2003). According to Jarrold (2003), individuals with ASD may have an underlying capacity to *understand* pretend, but fail to *engage in spontaneous* pretend. Several studies showing individuals with ASD as on par with controls at predicting the result of pretend sequences support the proposition of intact pretense comprehension in the ASD population (Jarrold et al., 1994; Kavanaugh and Harris, 1994).

In order to understand how pretend play varies across the range of ASD symptoms, it is useful to index variations in ASD traits in both TD and ASD populations (Russell-Smith et al., 2012), as ASD traits exist along a continuum in the general population (Constantino and Todd, 2003; Best et al., 2008). However, to date, little research has examined whether differences in pretend play production vary as a function of ASD symptoms dimensionally across TD and ASD populations. The sole exception is a recent study by Campbell et al. (2016), which found a negative correlation between ASD symptom severity and pretend play in toddlers. Moreover, little work has carefully explored the factors that contribute to spontaneous pretend among children with varying levels of ASD symptoms, the consideration of which will help better illuminate the factors that contribute to the development of the ability to pretend in children.

In addition to assessing how ASD symptoms might contribute to the ability to produce pretend, it is important to examine how various factors might directly influence different subtypes of pretend. Actions of pretend play often fall under one of three categories (Leslie, 1987): object substitution (OS; e.g., pretending a block is a car), attribution of pretend properties (APP; e.g., pretending dishes are dirty), and imaginary objects (IO; e.g., pretending to read a book when there is no book present). A small body of research suggests that it is not pretend as a whole, but certain types of pretend that are more challenging for individuals with ASD. For instance, Libby et al. (1998) demonstrated that children with ASD produced just as much OS as TD children, but produced significantly fewer acts of APP and IO, resulting in fewer acts of overall pretend compared to TD children. It is plausible that each of these categories of pretend may require slightly different child capacities and experiences (e.g., knowledge and understanding that a doll represents a human might lead a child to attribute the pretend property of talking, whereas knowledge and understanding that a doll is a vertical object that you can bang against things might lead a child to engage in OS and substitute the doll for a hammer), and may therefore represent categorically different styles of pretend play. Thus, assessing how certain predictors might influence pretend ability as a whole, as well as subtypes of pretend, may provide deeper insight into which elements of pretend (and associated mental representations) might contribute to difficulties with pretend among individuals with ASD. Moreover, researchers have not yet explored whether differences of pretend play subtypes produced would be evident among TD individuals with varying levels of ASD symptoms.

Associations between pretend play, ToM, and verbal ability are frequently found (Astington and Jenkins, 1995, 1999; Jenkins and Astington, 1996, 2000; Taylor and Carlson, 1997; Lewis et al., 2000; Bigham, 2010). Crucially, however, current research does not strongly support causal relations between engaging in pretend actions and the development of perspective-taking [theory of mind (ToM); Dore et al., 2015] and other developmental domains in TD children, calling for a greater rigor in clarifying relations of pretend play and these domains (Lillard et al., 2013). Therefore, carefully examining such links are helpful in understanding how pretend play normally develops, and how children with ASD symptoms might pretend differently.

ToM is the ability to understand others’ mental states and how other individuals might perceive the world (Leslie, 1987; Baron-Cohen, 1995; Wellman, 2014). Taylor and Carlson (1997) found a link between ToM ability and individual differences in pretend among TD children, suggesting that TD children with greater ToM ability may be able to produce more actions of pretend. These findings have since been supported longitudinally (Jenkins and Astington, 2000). That is, the ability to understand others’ perspectives may facilitate a greater ability to understand and produce pretend play. More recent evidence suggest that a third variable underpinning both ToM and pretend play, such as a more general symbolic capacity, may account for the link between ToM and pretend play (Lillard and Kavanaugh, 2014).

However, many individuals with ASD have difficulty understanding and interpreting others’ mental states (Baron-Cohen, 1995, 2001). Concurrently, while some children with ASD understand others’ actions of pretend, they still struggle to produce pretend (Jarrold, 2003). One explanation for these impaired abilities (ToM and pretend) among individuals with ASD might reflect difficulty in a symbolic substrate that underlies both pretend play and ToM development (e.g., Lillard and Kavanaugh, 2014).

Although, research supports a link between production of pretend and ToM ability (Taylor and Carlson, 1997) among TD children, this might represent a pathway typically used by TD individuals, and less often used by individuals with ASD. This might be because ToM appears not to develop organically among individuals with ASD. Instead, individuals with ASD may deliberately engage in a “hacked together” ToM, rather than intuitively engaging in a more “automatic” ToM (Dissanayake and Macintosh, 2003). The hacking hypothesis explains how individuals with high functioning ASD may pass ToM tasks via this less efficient, more deliberate route. Furthermore, those with ASD may engage in a “hacked together” ToM by learning specific rules that allow them to pass ToM tasks, rather than using social understanding and an understanding of other’s perspectives (Dissanayake and Macintosh, 2003). Given that intuitive, “automatic” ToM might not develop naturally among those with ASD, it is plausible that the presence of ASD symptoms may weaken the relation between ToM and pretend production.

On the other hand, pretend comprehension does appear to be related to ToM when considered among a large group of children including those with ASD, TD children, and children with intellectual disabilities (IDs; Bigham, 2010). However, pretense comprehension bears no relation to pretend production in TD children (Lillard and Kavanaugh, 2014). While Bigham’s (2010) findings suggest that ToM ability is linked to *understanding* pretend among individuals with ASD, no research has explored whether this link also exists when looking at the *production* of spontaneous pretend among this population. Additionally, no one has directly tested whether there would be a weaker relation between ToM ability and pretend play among individuals with more ASD symptoms compared to those with fewer symptoms. Furthermore, little research has explored differences in subtypes of pretend in relation to ToM ability.

A second suggested link with pretend play among TD children is with verbal ability (Lewis et al., 2000; Rutherford and Rogers, 2003; Bigham, 2010). Lewis et al. (2000) looked at the relation between language and pretend ability among verbal and non-verbal TD participants and assessed OS, APP, and IO. Lewis et al.’s (2000) findings indicated a relation between pretend play and language development. In addition, longitudinal studies have suggested that play and language may be developmentally related (e.g., Ungerer and Sigman, 1984; McCune, 1995; Lillard and Kavanaugh, 2014).

On the other hand, although verbal mental age seems to be correlated with pretend play production for both TD and children with other developmental disabilities (DDs) than ASD, such relation is not found in children with ASD (Rutherford and Rogers, 2003). This suggests that the link between verbal ability and pretend might be a pathway typically used by TD individuals and individuals with other DD, but one that individuals with ASD are less able to access. Individuals with ASD might be less able to access this pathway due to developmental deficits in communication, a diagnostic feature required for having an ASD (*DSM-5*, 2013). Similarly, those with more ASD symptoms might also experience some deficits in communication that make this pathway between verbal ability and pretend less accessible. Furthermore, given Rutherford and Rogers’s (2003) findings, one might predict that the relation between verbal ability and pretend would be weaker among TD children with greater levels of ASD symptoms; however, this has not been directly tested. Additionally, little research has explored differences in subtypes of pretend in relation to verbal ability.

If children with ASD are less able to use more typical pathways to pretend, like ToM and verbal ability, it is possible that individuals with ASD that are able to pretend use alternate pathways. Different factors may predict patterns of pretend in youth with ASD relative to TD and reflect the divergent developmental pathways used by children with ASD and TD children to arrive at the ability to produce actions of pretend. Two plausible factors are interest in and experience (familiarity) with specific toys. Although research studies have measured familiarity and interest to control for these factors when evaluating the effectiveness of pretend play interventions for youth with ASD (Murdock and Hobbs, 2010), little research has examined them as predictors of pretend play.

A child’s interest in an object can be characterized by repeated voluntary engagement with that object, with no outside encouragement (Hidi et al., 2004; DeLoache et al., 2007). As children explore different ways that they can interact with and play with those toys due to personal interest, pretend is one type of play that might result from repeatedly engaging with toys. Research has demonstrated that interest appears to drive and motivate the behaviors in which TD individuals choose to engage and the objects with which they chose to play (Hidi et al., 2004). However, these motivational aspects might play a minimal role in driving the pretend behavior of TD children, given pretending’s robust and seemingly spontaneous emergence in most TD children by the age of three (Lillard, 1993).

Presence of restricted, repetitive patterns of behavior, interests, or activities is a core feature of ASD (American Psychiatric Association [APA], 2013). Given that specialized interests (e.g., in toys or topics) are more common among ASD individuals (Baker et al., 1998; Vismara and Lyons, 2007), interest and motivation may play an especially crucial role in the play behaviors of youth with ASD (Koegel and Mentis, 1985). Even though children with ASD have been shown to engage in fewer actions of pretend and different subtypes of pretend (Libby et al., 1998), interest in objects might play an important role in initiating the actions of spontaneous pretend that these children can and do produce by motivating a child with ASD to repeatedly engage in actions of play with that toy. Due to a personal interest in a specific toy, children with ASD may have played with that toy frequently and already have an understanding of the typical functions of the toy. Hence, they might be more readily able to try out novel uses with it, such as pretend play. Such experiences may, in turn, facilitate basic metarepresentational capacities in a child with ASD.

Furthermore, familiarity with an object might also facilitate a child with ASD to produce actions of pretend. A child’s level of familiarity with an object is based on how often that child has seen or been exposed to that object (Hidi et al., 2004). The more children (either TD or ASD) are exposed to a certain object, the better they apperceive the characteristics of that object. Perhaps it is easier for a child with ASD to go beyond the percept to attribute abstract (pretend) characteristics to an object once they become more familiar with it. Conversely, as some youth with ASD tend to engage in repetitive behavioral routines with familiar objects (Leekam et al., 2011), they may in fact be more likely to engage in novel play routines (e.g., pretend) with less familiar objects. In this sense, familiarity could be seen to relate to more *or* less pretend in this population; crucially, though, it could plausibly be *more* related to pretend production in ASD relative to TD children.

Despite the proposed relation of interest in and familiarity with objects to spontaneous pretend play, and the theorized differences in this relation between TD and ASD youth, no previous research has explored this connection directly. Furthermore, no research has explored differences in subtypes of pretend in relation to interest in and familiarity with toys.

Using rigorous observational methods to assess spontaneous pretend play, the current study examined the relation between overall quantity and well-established subtypes of pretend play (OS, APP, and IO) spontaneously produced by TD and ASD children and measures of ASD symptoms, verbal ability, ToM, and interest in and familiar with presented toys. We hypothesized that (1) children reported to have more ASD symptoms would engage in less spontaneous pretend play overall, fewer acts of APP and IO, and comparable levels of OS relative to those with fewer ASD symptoms (Libby et al., 1998). Second, we hypothesized that (2a) both well-established (verbal ability and ToM) and novel (interest and familiarity) predictors of pretend would relate positively to overall pretend play. We also considered sub-hypotheses regarding the relation between ToM, verbal ability, and interest and familiarity, and subtypes of pretend. Given Libby et al.’s (1998) finding that individuals with ASD produced fewer acts of APP and IO, and the conjecture that certain pathways to pretend might be less (ToM and verbal ability) or more (interest and familiarity) accessible to individuals with more ASD symptoms, we hypothesized that (2b) better ToM and verbal ability would relate to more instances of APP and IO and that more interest in and familiarity with the presented toys would relate to more instances of OS. Furthermore, we hypothesized that (3a) ToM and verbal ability would show stronger relations to pretend quantity among individuals with fewer reported ASD symptoms compared to those with more symptoms. In a sub-hypothesis, we hypothesized that (3b) better ToM and verbal ability would result in more instances of APP and IO for those with fewer ASD symptoms. Finally, we hypothesized that (4a) levels of familiarity and interest in presented toys would show stronger relations to pretend quantity among individuals reported to have more ASD symptoms compared to those with fewer symptoms. In a sub-hypothesis, we hypothesized that (4b) more interest in and familiarity with the presented toys would result in more instances of OS for those with more symptoms of ASD compared to those with fewer symptoms.

## Materials and Methods

### Participants

Participants were thirty-four TD children (*M*_age_ = 6;1, *SD*_age_ = 2.0; 23 males) and six with ASD (*M*_age_ = 5;2, *SD*_age_ = 1.0; six males). Participants were recruited using the University of Virginia Babypool database, which is comprised of names and numbers of local Charlottesville families willing to be contacted to participate in research (see **Table 1** for demographic information). This study was carried out in accordance with the recommendations of University of Virginia Institutional Review Board for the Social and Behavioral Sciences, with written informed consent from parents of all subjects.

**Table 1:** Participant descriptive statistics (*N* = 40).

Variable	Mean *(SD)*	Range
Age	5.98 (1.46)	4.00–8.92
Sex	29 male	
Overall IQ	116.58 (15.46)	58–141
Social Communication Questionnaire	6.38 (7.12)	0–26
Verbal IQ	113.68 (15.17)	86–138
ToM	6.50 (3.56)	0–13
Familiarity	3.79 (0.57)	2.67–4.83
Interest	2.68 (0.66)	1.75–4.33
No Play	6.91 (4.13)	0.25–15.75
Non-Symbolic Play	8.93 (3.32)	3.25–16.25
Pretend Play	4.11 (3.45)	0–12.25
Pretend Play – Object Substitution	0.84 (1.22)	0–6.75
Pretend Play – Attribution of Pretend Properties	2.10 (3.05)	0–11.75
Pretend Play – Imaginary Objects	1.24 (2.41)	0–10.25


*ToM = Combined Theory of Mind score (Theory of Mind Scale plus Strange Stories).*

### Procedure

Each participant completed two visits. During their first visit, participants completed a measure of cognitive ability, including verbal ability (the Kaufman Brief Intelligence Test 2; KBIT-2; Kaufman and Kaufman, 2004). Parents completed a measure of ASD symptoms throughout their child’s development [Social Communication Questionnaire (SCQ); Rutter et al., 2005], a standard developmental history form, and a questionnaire regarding their child’s experience with and interest in specific toys.

At the beginning of the second visit (usually completed by the same research assistant as visit 1), ToM measure(s) were administered, followed by two 5-min free-play sessions with toys. One play session involved a set of six conventional objects (toy car, female doll, male doll, pan, spoon, and bowl) and the other involved a set of six “junk” objects (piece of string, piece of cardboard, butter tub, margarine tub, empty spool, and empty matchbox), consistent with the work of Libby et al.’s (1998) study. Both conventional and junk objects were used given that “high structured” conventional objects have been shown to elicit more pretend play than “low structured” junk objects (McLoyd, 1983). Having both toy sets allowed for gathering more data regarding how children pretend with different toys, and allowed for estimation of each child’s “average” spontaneous pretending across situations that may tend to elicit more or less pretense. The order in which object sets were presented was randomized.

When introducing a toy set, the six objects were arranged in a semicircle around the child, ensuring that each toy had an equal opportunity of being selected (Servin et al., 1999). While placing the toys, the research assistant recited the following script (similar to McLoyd, 1983; Baron-Cohen, 1987): “I have some work to finish up. I will be back in a few minutes. Here, are some toys for you to play with while I’m working. You can do anything you like with them.” After 5 min of free-play with the first set of objects, the next set was introduced in the same manner, and the procedure repeated. All play sessions were videotaped to allow for independent blinded subsequent coding of play content by separate raters.

### Measures

#### SCQ (Rutter et al., 2005)

This parent-report questionnaire is a widely used screener for ASD. This measure examines the presence or absence of specific ASD symptoms across a child’s development thus far, and was used to compare ASD symptom levels across participants. The subscales of the SCQ are reciprocal interactions (example item: Does your child have any particular friends or a best friend?), communication (example item: Did your child ever spontaneously point at things around him/her just to show you things [not because she/he wanted them]?), and restrictive repetitive behaviors (example item: Did your child seem unusually interested in the sight, feel, sound, taste, or smell of things or people?; Rutter et al., 2005). These subscales represent the three core deficits among individuals with ASD. SCQ scores can range from 0 to 40 and scores above the threshold of 15 suggest a high likelihood of meeting criteria for ASD.

#### Theory of Mind Scale (ToM Scale; Wellman and Liu, 2004)

This ToM Scale was used, because it is a standardized instrument designed to measure ToM development among TD individuals up through the mental age of 5 (Peterson et al., 2005). To maximize the sensitivity of these scales to diverse forms of ToM we used the original six-items version. The scale is comprised of six tasks, with each task increasing in level of difficulty. The tasks are: diverse desires, diverse beliefs, knowledge access, contents false belief, explicit false belief, and real-apparent emotion (Wellman and Liu, 2004). All six tasks were administered due to the finding that no task alone can account for the progressive development of ToM capabilities (Wellman and Liu, 2004). Participants were awarded zero points for incorrect answers and one point for correct. Total points awarded could range from 0 to 6.

#### Strange Stories (Happé, 1994)

This advanced ToM battery is able to effectively measure ToM capabilities among TD individuals ranging in age from 5 to 12 years old (O’Hare et al., 2009). There were 10 stories, nine of which were measures of ToM ability, and one was a control story that only asked questions regarding physical events to ensure that there was no comprehension deficit. The stories were either read aloud to the participant by the experimenter or the child read the story aloud. Administration of Strange Stories allowed for a deeper examination of a child’s ToM development by looking at whether or not the child was able to provide mental state explanations for why a story’s character might have acted a certain way (Happé, 1994). In order to test for higher levels of ToM, Strange Stories was administered to any participant that passed all six of the tasks presented in the Wellman and Liu (2004) ToM scale. Participants received a score of 1 (successfully provided the correct mental state explanation for a character’s behavior) or 0 (failed to provide correct mental state explanation) for each story, yielding overall scores ranging from 0 to 9 on this measure. Scores on the ToM Scale and Strange Stories were summed to create a composite ToM score for youth across the given age range, ranging from 0 to 15.

#### KBIT-2 (Kaufman and Kaufman, 2004)

The KBIT-2 is a measure of verbal IQ, non-verbal IQ, and Full Scale IQ scores. It produces standard scores (*M* = 100, *SD* = 15). The verbal IQ score was used to assess participant’s verbal ability and ensure that each participant was of normally developed intelligence (IQ > 85) and verbal ability (verbal IQ > 85). The benefit of using the KBIT-2 is that it is faster to administer than the more common Wechsler intelligence scales, while still measuring both verbal and non-verbal cognitive functions and providing a composite IQ score (Naugle et al., 1993). Furthermore, research findings (Naugle et al., 1993) indicate that scores from the KBIT are comparable to the Wechsler scales in this age range, and construct validity was supported.

#### Toy Survey

Parents completed a survey in which they indicated their child’s interest in and familiarity with each of the toys presented during the play sessions. Parents rated interest and familiarity for all of the toys on a 1 (*not at all*) to 5 (*extremely)* scale. This resulted in 12 ratings of familiarity and 12 ratings of interest (given the six junk and six conventional toys). These ratings were averaged in order to get an overall measure and gross approximation of each participant’s familiarity and interest with these sets of toys.

#### Pretend Play Coding Scheme

The pretend play coding scheme was designed to code for different types of play (No Play, Non-Symbolic Play, Pretend Play) and, if pretending, subtype (OS, APP, IO; Libby et al., 1998). No Play meant that a child was engaging in various types of behavior, but that he or she was not playing (e.g., not attending to objects, labeling objects, and looking at objects without acting on them). Any type of play that could not be categorized as one of the three types of pretend was coded as Non-Symbolic Play (e.g., piling and stacking objects, spinning objects, tossing objects, and banging objects). OS was defined as clearly using an object as if it was another *specific* item (e.g., using the doll as a spoon, using the car as a piece of food). APP was defined as indicating the presence of features (i.e., color, size, abilities to talk) to an object that deviated from the true features an object actually had. These features could represent actual characteristics the object *could* have or *imaginary* characteristics (e.g., walking the female or male, and claiming the toy pan was hot). IO was defined as acting as if an actual item that was *not* present in the room was in fact present. IO required the behavior to involve the presented toys (i.e., could not involve talking to an imaginary friend), and be explicit such that the coder could clearly identify each absent object (e.g., eating imaginary food, stirring “something” in pan or bowl). These definitions were chosen to maximize clarity that target behaviors were observed, yielding conservative estimates of each play type.

Pairs of coders, blind to each other’s scores as well as the scores of the observed child on other measures, watched 5-min play sessions and coded the play in 15-s intervals, similar to the procedure of Libby et al. (1998). The coding team consisted of three undergraduate students naïve to study hypotheses. Over a 2-month period, the team was trained by reading the pretend play coding manual, attending weekly meetings, practicing coding using training tapes, and reviewing and discussing specific training intervals. ICCs were calculated to assess reliability according to standards specified by Cicchetti (1994). Coders were “certified” for coding once their ratings, as a group, achieved acceptable scale level interrater reliability [ICC(2,4) > 0.60] relative to master codes on 20 separate practice tapes of child interactions with a variety of toys. Once coding began, reliability assessments were performed and discussed at weekly meetings to minimize coder drift (Margolin et al., 1998).

All sessions were observed and double-coded for play type (no play, non-symbolic play, pretend play) and, if pretending, subtype (OS, APP, IO; Libby et al., 1998), yielding 62 pairs of ratings. For each 15-s interval, play was coded for the highest level of play in which participants engaged (From lowest to highest; No Play, Non-Symbolic Play, Pretend Play). If Pretend Play was selected, the subtype of pretend was also coded. Subtype of pretend was based on which type of pretend best characterized the interval. Each participant could have engaged in any type of play, or subtype of pretend, yielding scores of 0 to 20 for each play type and subtype, given the 15-s intervals and the 5-min play session. We averaged across the two 5-min play sessions (conventional and junk) in order to obtain a sample of spontaneous pretend from situations shown to elicit lesser and greater quantities and qualities of pretend (McLoyd, 1983). This was then used to determine each child’s “average” ability to produce actions of pretend. Interrater reliability ICC(1,2) was excellent for No Play (0.85), Non-Symbolic Play (0.83), Pretend Play (0.90), APP (0.89), and IO (0.97), and was good for OS (0.63) (Cicchetti, 1994).

### Data Analytic Plan

We first used descriptive analyses to assess amount of play types and pretend play subtypes produced (No Play, Non-Symbolic Play, Pretend Play, OS, APP, and IO), number of ASD symptoms, verbal ability, ToM ability, and interest in and familiarity with the toys. We then used bivariate correlations to explore relations between continuous variables. To test hypothesis 1, that children with more ASD symptoms would engage in less spontaneous pretend overall, fewer acts of APP and IO, and more acts of OS relative to lower scoring participants, we examined the correlations between play types and pretend subtypes compared to SCQ scores.

To test hypothesis 2, that both well-established (verbal and ToM abilities) and novel (interest and familiarity) factors would relate positively to amount of overall pretend play, we looked at the correlations between each factor and overall pretend. To examine the sub-hypothesis that better ToM and verbal ability would result in more instances of APP and IO and that more interest in and familiarity with presented toys would result in more instances of OS, we examined correlations between these predictors and the respective pretend subtypes.

Our third hypothesis was that ToM and verbal ability would show stronger relations to overall pretend among individuals with fewer ASD symptoms compared to those with more symptoms. The sub-hypothesis was that better ToM and verbal ability would predict more instances of APP and IO for those with fewer ASD symptoms compared to those with more symptoms. To test these hypotheses, we ran hierarchical multiple regressions predicting overall pretend, APP, and IO, with age and ASD status on step 1, predictors (SCQ scores, ToM, verbal ability) on step 2, and the interactions between SCQ scores and age, ToM, and verbal ability on step 3. Interactions were investigated with *post hoc* probing (Holmbeck, 2002).

Our fourth hypothesis was that levels of interest in and familiarity with presented toys would show stronger relations to overall pretend among individuals with more ASD symptoms compared to those with fewer symptoms. The sub-hypothesis was that more interest in and familiarity with the presented toys would result in more instances of OS for those participants with more symptoms of ASD compared to those with fewer symptoms. To test these hypotheses, we used the same overall pretend regression model as for the third hypothesis (and ran an identical model predicting OS), except we examined interactions between interest, familiarity, and SCQ scores. Interactions were again probed. For hypotheses 1, 3, and 4, significant effects were probed by re-running analyses with the SCQ score replacing each of its 3 subscales to determine which, if any, were driving the effect.

## Results

### Descriptives

**Table 1** presents descriptive statistics. Participants displayed normal intelligence and verbal ability, and a wide range of interest in and familiarity with the toys. Participants also displayed developmentally-appropriate ToM ability. Participants ranged from having a history of 0 ASD symptoms to 26 ASD symptoms; 15 symptoms is the screening cutoff for ASD. Play scores indicate that participants’ play was characterized by No Play about a third of the time, Non-Symbolic Play about half the time, and Pretend Play about a fifth of the time. However, most participants (±1 *SD*) engaged in 0–7 instances of Pretend Play. In terms of subtypes of pretend, APP was the most common and although OS was rare, it still made up roughly a quarter of the observed instances of Pretend Play.

### Bivariate Correlations

**Table 2** presents correlations between continuous variables. Older participants had better ToM, fewer instances of No Play, and more instances of Non-Symbolic Play and APP. Older participants also displayed more familiarity with the toys.

**Table 2:** Correlations among continuous variables.

	Age	IQ	SCQ	VIQ	ToM	FAM	INT	NP	NSP	PP	PP-OS	PP-APP	PP-IO
Age	1	0.27	-0.31	0.21	0.57**	0.38*	-0.21	-0.49**	0.36*	0.26	-0.08	0.289	0.03
IQ		1	-0.38*	0.81**	0.52**	0.15	0.10	-0.20	0.13	0.11	-0.14	0.13	0.06
SCQ			1	-0.53**	-0.42**	-0.46**	-0.20	0.34*	-0.41**	-0.01	-0.01	0.19	-0.22
VIQ				1	0.57**	0.19	0.16	-0.31	0.37*	0.01	-0.05	-0.06	0.12
ToM					1	0.11	-0.08	-0.53**	0.41**	0.24	-0.14	0.32*	0.03
FAM						1	0.24	-0.12	0.15	-0.003	0.14	-0.25	0.23
INT							1	0.10	0.11	-0.24	0.37*	-0.25	-0.20
NP								1	-0.59**	-0.63**	-0.23	-0.41**	-0.27
NSP									1	-0.26	0.05	-0.18	-0.16
PP										1	0.22	0.68**	0.46**
PP- OS											1	-0.10	-0.03
PP- APP												1	-0.25
PP- IO													1


*^∗^*p* < 0.05, two-tailed; ^∗∗^*p* < 0.01, two-tailed. SCQ, Social Communication Questionnaire; VIQ, Verbal IQ; FAM, Familiarity; INT, Interest; ToM, Combined Theory of Mind score (Theory of Mind Scale plus Strange Stories); NP, No Play; NSP, Non-Symbolic Play; PP, Pretend Play; PP-APP, Pretend Play-Attribution of Pretend Properties; PP-OS, Pretend Play-Object Substitution; PP-IO, Pretend Play-Imaginary Objects.*

Participants with higher overall IQ and verbal IQ had less ASD symptoms and better ToM. Participants with higher verbal IQ (only) had more Non-Symbolic Play.

More ASD symptoms predicted less ToM, less familiarity with the toys, more No Play, and less Non-Symbolic Play. Among all participants, better ToM predicted less No Play and more Non-Symbolic Play. Finally, more interest in the toys predicted more OS. Subtypes of pretend play were *not* significantly correlated with each other.

### Hypothesis 1 –ASD Symptoms and Pretend

No significant correlations were found between SCQ scores and the amount of overall pretend produced or subtypes of pretend produced (**Table 2**).

### Hypothesis 2 – ToM, Verbal Ability, and Interest and Familiarity and Pretend

Theory of mind, verbal ability, and interest and familiarity were not shown to be predictors of overall pretend. However, better ToM predicted more APP to the toys and more interest in the toys predicted more OS with the toys. Familiarity and verbal ability were not shown to be predictors of any pretend play subtypes (**Table 2**).

### Hypothesis 3 –ToM, Verbal ability, ASD Symptoms and Pretend

**FIGURE 1:**
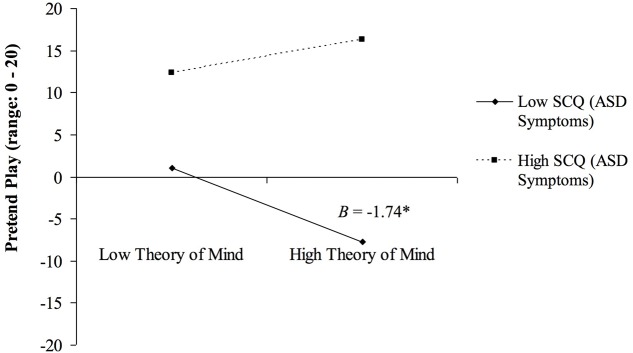
**Values below 0 are model-predicted values.** Interaction between Social Communication Questionnaire [SCQ; i.e., history of many versus few autism spectrum disorder (ASD) symptoms] and theory of mind (ToM) Scale scores predicting amount of observed pretend play. Among those with low SCQ, a negative relation was found between ToM and pretend play. ^∗^*p* < 0.05. Low = 1 SD below sample mean. High = 1 SD above sample mean.

Significant interactions were found between ASD symptoms and VIQ (*B* = -0.014, *p* = 0.034), and ASD symptoms and ToM (*B* = 0.18, *p* = 0.004) in predicting overall pretend play (**Table 3**). An interaction was found between ASD symptoms and ToM (*B* = 0.10, *p* = 0.021) in predicting IO (**Table 4**). Probing indicated a negative relation between ToM and pretend play among those with low SCQ (*B* = -1.74, *p* < 0.05; **Figure 1**) and a marginally negative relation between VIQ and pretend play among those with high SCQ (*B* = -0.13, *p* < 0.10). Effects were consistent across subdomains of the SCQ, and were reduced when excluding ASD participants.

**Table 3:** Hierarchical multiple regression predicting amount of observed spontaneous pretend play.

	Model 1			Model 2			Model 3		
			
	*B*	*SE B*	β	*B*	*SE B*	β	*B*	*SE B*	*B*
Age	0.69	0.38	0.29	0.72	0.43	0.30	0.73	0.51	0.31
ASD status	0.83	1.55	0.09	0.52	4.43	0.06	-1.78	4.71	-0.19
SCQ VIQ				0.01	0.20	0.02	0.80	0.76	1.64
ToM				0.00	0.05	0.01	0.07	0.06	0.29
				-0.12	0.53	-0.05	-1.61	0.67	-0.60^∗^
SCQ × age							0.02	0.06	0.16
SCQ × VIQ							-0.01	0.01	-2.72^∗^
SCQ × ToM							0.18	0.06	1.30^∗∗^
Total *R^2^*		0.08			0.08			0.35	
*F* for *ΔR^2^*		1.63			0.02			4.23^∗^	


*^∗^*p* < 0.05, ^∗∗^*p* < 0.01. ASD status, binary variable, confirmed using Autism Diagnostic Observation Schedule (Lord et al., 1999); SCQ, Social Communication Questionnaire (Rutter et al., 2005); VIQ, Verbal Intelligence Quotient (from KBIT-2; Kaufman and Kaufman, 2004); ToM, Theory of Mind Scale (Wellman and Liu, 2004).*

**Table 4:** Hierarchical multiple regression predicting imaginary objects subtype of pretend play.

	Model 1			Model 2			Model 3		
			
	*B*	*SE B*	β	*B*	*SE B*	β	*B*	*SE B*	*B*
Age	-0.02	0.28	-0.01	0.09	0.28	0.06	0.3	0.36	0.20
ASD status	-1.19	1.11	-18.0	-0.42	2.90	-0.06	-0.37	3.35	-0.06
SCQ VIQ				-0.09	0.13	-0.27	0.18	0.54	0.54
ToM				0.02	0.03	0.11	0.04	0.04	0.23
				-0.83	0.35	-0.44	-1.65	0.48	-0.88^∗∗^
SCQ × age							-0.04	0.04	-0.56
SCQ × VIQ							-0.004	0.005	-1.17
SCQ × ToM							0.10	0.04	1.03^∗^
Total *R^2^*		0.03			0.19			0.32	
*F* for *ΔR^2^*		0.59			2.28			1.99	


*^∗^*p* < 0.05, ^∗∗^*p* < 0.01. ASD status, binary variable, confirmed using Autism Diagnostic Observation Schedule (Lord et al., 1999); SCQ, Social Communication Questionnaire (Rutter et al., 2005); VIQ, Verbal Intelligence Quotient (from KBIT-2; Kaufman and Kaufman, 2004); ToM, Theory of Mind Scale (Wellman and Liu, 2004).*

### Hypothesis 4 – Interest and Familiarity, ASD Symptoms and Pretend

**FIGURE 2:**
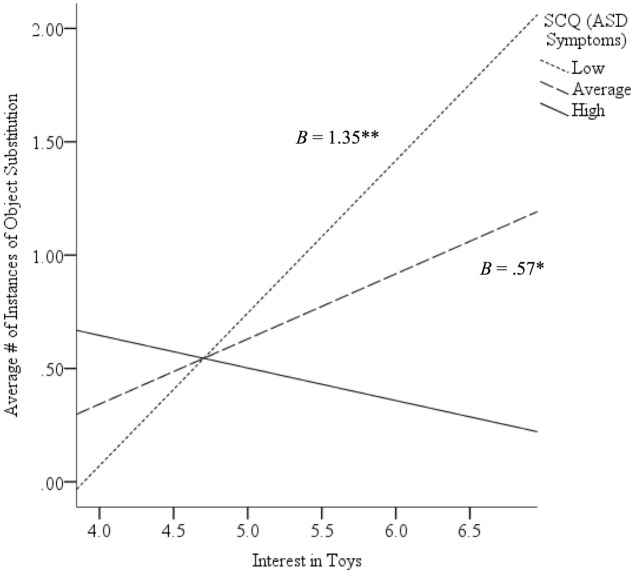
**Relation between level of interest in toys and observed instances of object substitution (OS) based on high, average, or low scores on the Social Communication Questionnaire [SCQ; i.e., autism spectrum disorder (ASD) symptoms].** While there was a positive relation between interest and quantity of OS for those with a history of few or average ASD symptoms, this relation was not found for those with a history of more ASD symptoms. ^∗^*p* < 0.05, ^∗∗^*p* < 0.01. Low = 1 SD below sample mean. Average = sample mean. High = 1 SD above sample mean.

There was no interaction between SCQ scores and familiarity in predicting overall pretend play or OS (both *p* > 0.56), or between SCQ scores and interest in predicting overall pretend play (*p* = 0.09). However, there was an interaction such that the relation between interest and OS differed based on participants’ SCQ scores (**Table 5**, Model 2). *Post hoc* probing of this interaction suggests that there was a positive relation between interest and OS when SCQ scores were low (*B* = 1.35, *p* < 0.01) and average (*B* = 0.57, *p* < 0.05; **Figure 2**).

**Table 5:** Hierarchical multiple regression predicting object substitution subtype of pretend play.

	Model 1			Model 2		
		
	*B*	*SE B*	β	*B*	*SE B*	β
SCQ	-0.01	0.04	-0.05	-0.20	0.43	-1.16
ToM	-0.03	0.07	-0.08	0.05	0.10	0.13
VIQ	-0.01	0.02	-0.10	-0.03	0.03	-0.36
FAM	0.12	0.39	0.06	-0.16	0.53	-0.7
INT	0.66	0.31	0.36^∗^	1.45	0.51	0.79^∗∗^
SCQ × ToM				-0.01	0.02	-0.32
SCQ × VIQ				0.004	0.003	1.95
SCQ × FAM				0.08	0.11	1.52
SCQ × INT				-0.16	0.07	-2.21^∗^
Total *R^2^*		0.40			0.54	
*F* for *ΔR^2^*		1.31			1.35	


*^∗^*p* < 0.05, ^∗∗^*p* < 0.01. ASD status, binary variable, confirmed using Autism Diagnostic Observation Schedule (Lord et al., 1999); SCQ, Social Communication Questionnaire (Rutter et al., 2005); VIQ, Verbal Intelligence Quotient (from KBIT-2; Kaufman and Kaufman, 2004); ToM, Theory of Mind Scale (Wellman and Liu, 2004); FAM, Familiarity; INT, Interest; SCQ × FAM, Interaction between familiarity with toys and SCQ; SCQ × INT, Interaction between interest in toys and SCQ.*

### *Post hoc* Analyses

**FIGURE 3:**
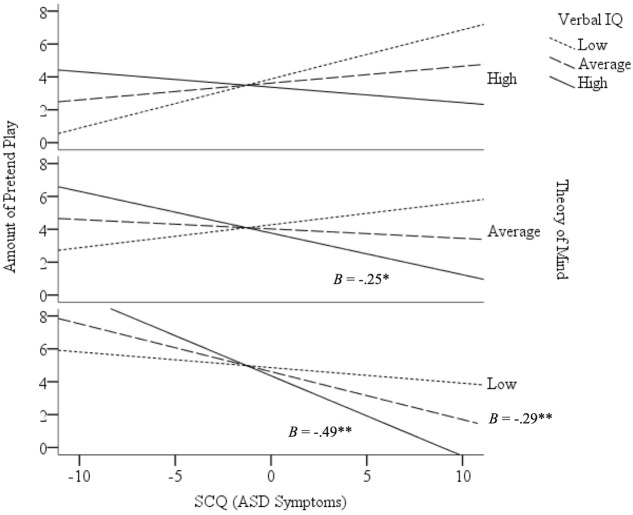
**Simultaneous moderation by ToM and verbal ability (VIQ) on the relation between ASD symptoms and pretend play.** More ASD symptoms related to less pretend play production when ToM was low and VIQ was average or high, and when ToM was average and VIQ was high. All other relations were non-significant. The negative relation between ASD symptoms and pretend play was especially pronounced among those with low ToM and high verbal ability. *p < 0.05, **p < 0.01. Low = 1 SD below sample mean. Average = sample mean. High = 1 SD above sample mean.

Previous studies have reliably found that VIQ and ToM each play a role in pretend play production. In the present study, interactions were found between ASD symptoms and VIQ and between ASD symptoms and ToM, indicating a negative relation between VIQ and pretend play among those with more ASD symptoms and a negative relation between ToM and pretend play among those with fewer ASD symptoms. Given the surprising relation in the opposite directions between ASD symptoms and pretend play when taking into account these variables separately, characterizing how these processes impact pretend play outcomes may require one level of complexity higher than typically analyzed. That is, jointly operating to influence the relation between ASD symptoms and pretend play production. Further probing of these effects while controlling for age revealed that the effect of ASD symptoms on pretend play was moderated *simultaneously* (i.e., double moderation) by VIQ (*B* = -0.01, *p* = 0.03) and ToM (*B* = 0.18, *p* = 0.003). *Post hoc* analyses showed that the negative relation between ASD symptoms and pretend play was present *only* in those with low ToM and average or high VIQ (*B* = -0.29, *p* = 0.005 and *B* = -0.49, *p* = 0.006, respectively), as well as those with average ToM and high VIQ (*B* = -0.25, *p* = 0.04; **Figure 3**). That is, lower ToM and higher VIQ both related to less pretend play production among children with more ASD symptoms.

## Discussion

The current study examined ASD symptoms, as well as ToM, verbal ability, and interest in and familiarity with presented toys, as potential predictors or moderators of overall pretend and subtypes of pretend play spontaneously produced by children.

First, we hypothesized that measures of ASD symptoms would correlate with overall pretend and subtypes of pretend spontaneously produced, such that those with more ASD symptoms would engage in fewer actions of overall pretend, less APP and IO, and more OS. However, findings did not support this hypothesis, as ASD symptoms did not correlate with overall pretend or subtypes of pretend. Although, previous studies have shown that the overall pretend and subtypes of pretend differ between TD individuals and individuals with ASD (Libby et al., 1998), these differences in pretend production were not seen across children with varying degrees of ASD symptoms. This suggests that although the number of ASD symptoms may vary among TD children, this variance alone did not result in differences in either the amount of overall pretend or subtypes of pretend produced. It may be that a sufficient quantity of ASD-related deficits is required before ASD symptoms influence pretend ability.

Second, we hypothesized that established (ToM, verbal ability) and novel (interest in and familiarity with the presented toys) predictors of pretend would positively correlate with overall pretend. Additionally, we hypothesized that better ToM and verbal ability would result in more instances of APP and IO, and that more interest in and familiarity with the presented toys would result in more instances of OS.

While previous studies have found compelling links between ToM ability and pretend (Taylor and Carlson, 1997), the current study found that, whereas better ToM was *not* correlated with overall pretending, it *was* linked to more instances of APP. This finding suggests that ToM ability might contribute more to engaging in and understanding certain types pretend. Furthermore, it is interesting that this link was only found with APP and not IO or OS. When engaging in actions of APP, children explore what else an object can do. Thus, both ToM and APP involve ascribing novel capacities to people (i.e., ability to know something they might otherwise not) and to objects (i.e., the ability to do something that they might otherwise not). This suggests that rather than emanating from a basic *metarepresentation* ability (Leslie, 1987), both ToM ability and the ability to attribute pretend properties may stem from a common *metaattribution* ability.

Additionally, more interest in the toys was linked to more OS. Interest in the toys was explored as a novel predictor of pretend and pretend subtypes on the basis that children might be more motivated to engage in higher levels of play, like pretend, if they were more motivated to play with the presented toys. Past literature suggests that interest in objects drives the play behaviors in which children choose to engage (Hidi et al., 2004). The finding that interest correlated with more OS suggests that those with more interest in the presented toys may engage in more *holistic* exploration of them. Holistic exploration means that children explore the characteristics of the entire object (e.g., toy car) rather than focusing on individual features of that object (e.g., wheels). OS may reflect such holistic exploration, as it is a subtype of pretend that involves exploring what else the whole object can be (in contrast to the other two types, which involve exploration of what else an object can do [APP] and what outside associations can be made [IO]). Whole OS is also more likely to be related to its shape (Smith and Jones, 2011), which translates to sensitivity to its spatial characteristics. This finding also indicates that interest in toys might play a previously unexamined role in the development of the ability to pretend among children. Interestingly, our results suggest that although interest may relate to the ability to engage in OS, the influence of interest may vary across different ASD symptomatology. Contrary to our hypothesis, while more interest in the presented toy is related to more OS among those with few or average ASD symptoms, no such relation was found between interest and OS among those with more ASD symptoms. High interest in specific toys may be related to broader cognitive rigidity (e.g., insistence on sameness, resistance to change) in children with greater ASD symptomatology (Carcani-Rathwell et al., 2006) and may interfere with the ability to consider the toy as a different object.

Thus, ToM and interest in the toys predicted distinct, hypothesized subtypes of pretend. These findings highlight the importance of directly examining and understanding different types of pretend. This, in conjunction with our finding that pretend subtypes were not significantly correlated *with each other*, further stresses the importance of dissociating subtypes of pretend.

Conversely, although previous literature has suggested a link between verbal ability and pretend among TD individuals (Lewis et al., 2000; Rutherford and Rogers, 2003) findings from the current study indicated that verbal ability did not predict overall pretend or subtypes of pretend produced. However, previous literature has used much younger samples of TD participants; for example, all of Rutherford and Rogers’ (2003) participants were younger than 4 years old and Lewis et al.’s (2000) ranged in age from 1 to 6. Furthermore, our findings are consistent with previous literature that did not find a relation between verbal ability and pretend production among certain samples, such as individuals with ASD (Rutherford and Rogers, 2003). This suggests that although verbal ability may relate to the ability to produce actions of pretend it is possible that verbal ability’s influence varies across populations and diminishes with age. Additionally, the current study used a more conservative measure to code pretend in order to ensure that coders were certain that each instance of pretend coded was in fact pretend. Thus, it is possible that the link between verbal ability and pretend disappears when more rigorous, conservative measures are used to assess pretend play (Lillard et al., 2013).

Familiarity with the toys was also not shown to predict either overall pretend or pretend subtypes. Whereas past literature has suggested that more familiarity with toys indicates better knowledge and understanding of the characteristics of those toys (Hidi et al., 2004), the current study’s findings suggest that perhaps familiarity does not induce participants to engage in pretend with presented toys. That is, knowing what a toy is may not be sufficient to prompt greater exploration of it as an object with which to pretend.

Third, we hypothesized that the links between ToM, verbal ability and overall pretend produced would be stronger for those with fewer ASD symptoms compared to those with more symptoms. We also hypothesized that better ToM and verbal ability would result in more instances of APP and IO for those with fewer ASD symptoms compared to those with more symptoms. However, no significant interaction was found. Perhaps this was because the current study looked at ASD symptoms within TD children rather than conducting a direct comparison between TD children and children with ASD. Again, it may be that it takes a sufficient quantity of ASD-related deficits before these ASD symptoms are able to highlight distinct differences in the way ToM and verbal ability influence production of pretend.

Finally, we hypothesized that the links between interest in and familiarity with toys and overall pretend produced would differ based on ASD symptoms, such that these relations would be stronger for those with more ASD symptoms compared to those with fewer symptoms. We also hypothesized that more interest in and familiarity with the presented toys would result in more instances of OS for those with more symptoms of ASD compared to those with fewer symptoms. Although we again found no effect for familiarity, there was an interaction such that the relation between interest and OS differed based on SCQ scores. Contrary to our hypothesis, while there was a positive relation between interest and OS across the sample, this relation was stronger when SCQ scores were low. As noted by the weak central coherence (WCC) theory, youth with ASD (as well as TD youth with more ASD symptoms) tend to demonstrate a detail-focused cognitive style, and have consequent difficulty seeing the “big picture” in various contexts (Happe and Frith, 2006). More holistic exploration (i.e., OS) with a toy may be seen as a play-based behavioral indicator of such “big picture” engagement. Thus, this finding is consistent with the WCC theory, as it suggests that individuals with more ASD symptoms might tend toward relatively more detail-focused engagement with toys rather than the holistic type of play reflected in OS. This is also consistent with recent work (Russell-Smith et al., 2012) indicating a relation between detail-focused cognitive style and ASD-like impairments in social development in TD individuals. This result further highlights the importance of considering subtypes of pretend play when examining its relation to cognitive development, and future work should explore this intriguing link between OS and features related to ASD.

Additionally, *post hoc* analyses indicated that developmental delays in communication, but not reciprocal interaction or restrictive repetitive behaviors, seemed to drive this relation. This finding, along with the finding of no interaction between verbal ability and SCQ scores in predicting pretend, suggests that historical (rather than current) impairments in development of communication ability might prevent children from being able to connect their interest in toys to pretending in very sophisticated ways with the toys (i.e., OS). However, while the interaction effect for communication was larger than either reciprocal interaction or restrictive repetitive behaviors, it is important to note that the interaction was only marginally significant. Thus, it appears that aggregate ASD symptoms appear to best account for this effect.

Results suggest that, while ASD symptoms are not a predictor of spontaneous pretend play on their own, there are unexpected negative relations between ToM and pretend play associated with ASD symptoms, as well as between VIQ and pretend play associated with ASD symptoms. Given the reliable findings in previous studies of relations between each of these variables, it is important to consider the role that ToM and VIQ can play *simultaneously* in pretend play production in children with varying degree of ASD symptoms. Specifically, the predicted negative relation between ASD symptoms and pretend play was more pronounced in those with low ToM ability and high verbal ability. Previous inconsistent findings in relations of VIQ, ToM, and pretend play may be related to variability in ASD symptoms in sampled populations, and it is notable that the relation between ASD symptoms and pretend play was clarified in the current study only by examining effects of ToM and VIQ together on such relation. Whereas, ability to pretend are both require the ability to be metarepresentational (Leslie, 1987), verbal ability may not require metarepresentational capacity (Breheny, 2001) and may involve non-metarepresentational diversion of cognitive resources. For example, this may help explain the pattern of play seen in children with ASD who presents with good verbal ability (e.g., those who are hyperlexic or hyperverbal; Grigorenko et al., 2002) but show poor ToM and show difficulties with pretend play production.

### Limitations and Future Directions

Although this study provided a novel inquiry into predictors of spontaneous pretend play in children with and without ASD, there are (as always) several limitations. First, the sample size was relatively small, and while this study examined ASD symptoms in TD youth and ASD youth, the ASD sub-sample was very small. Thus, it cannot strongly support any contention that observed findings relate to ASD symptoms and not other factors (e.g., delays in communication development) to which the employed measures may be sensitive. Likewise, if deficits in pretend play are truly specific to those who meet diagnostic criteria for ASD (i.e., are pathognomonic), then this study would be underpowered to detect the hypothesized severity-related effects, even if they were present. Thus, future studies should explore the same predictors (ToM, verbal ability, familiarity, and interest) with a bigger sample with greater variability in ASD symptoms in order to reveal the relations between these variables more clearly.

Second, the age of participants was fairly broad, ranging from age 4 to age 8. Indeed, the types of play that children engage in across these ages do tend to vary (Johnson, 1998). Because of the relatively wide age range, and consequently diverse forms of play emerging across the age range, there is a possibility that different play behaviors were categorized similarly by the coding system. Future studies should examine narrower age ranges in larger samples in order to develop richer picture of how and what pretend play looks like in this paradigm at specific points in development.

Third, we used a measure that provided only a rough approximation of past history of ASD symptoms. The SCQ is a measure of historical ASD symptoms, not present. Thus, future studies should employ continuous measures of contemporaneous ASD symptoms, such as the Autism Spectrum Quotient –Children’s Version (AQ-Child; Auyeung et al., 2008) or the Social Responsiveness Scale-2 (SRS-2; Constantino and Gruber, 2012) to obtain a current measure of ASD symptoms.

Fourth, the pretend play coding scheme was a novel system for coding pretend play and play type. While the system proved to be reliable and rigorous, it is difficult to make direct comparisons to past studies that also looked at production of pretend play but used different measures. Future studies should use similar systems when coding actions of pretend play and play type with toys to determine if similar findings occur. Future studies should also examine differences in pretend play patterns as driven by interest and familiarity in a specific toy. This will not only serve as a validity check for the procedure itself (i.e., do children whose parents identify dolls as toy with highest interested and familiarity show pretend play with dolls the most) but also augment the utility of this novel paradigm.

Finally, the system we created for coding pretend play and play type also used a very conservative estimate of pretend. We chose to take a more conservative approach to coding pretend to ensure that coders were absolutely certain an action with the toys was pretend before coding it. However, by taking this more conservative approach, it is possible that certain instances of pretend were missed, especially for subtypes of pretend with low frequency of occurrence like OS. Taken together, this study would benefit from replication to further support the unexpected findings.

### Clinical and Theoretical Implications

This was the first study to examine the relation between ASD symptoms in children across typical and atypical development and observed spontaneous pretend play. It was the first to examine the relation between novel predictors (interest in and familiarity with presented toys) and pretend play. Finally, it was one of the first to carefully consider predictors of differences in subtypes of pretend.

One of the strengths of the current study was that it presented a new system for coding and assessing pretend play and play types with different toys. This coding system, while novel, proved to be a rigorous, conservative, reliable measure of play type using blinded raters (a crucial, under-represented approach in this literature; see Lillard et al., 2013), and may prove to be a useful measure for future research studies aiming to code play type with objects. Indeed, based on the methodological strengths of this measure, these results can be seen to shed new light on the prediction of pretend.

This study suggests that ASD symptoms are *not*, on their own, a predictor of spontaneous play with toys in children. However, our results highlight the importance of how varying degree of ASD symptoms may interact with child’s verbal ability and ToM to influence production of pretend play. It also suggests that abnormal play behaviors may be truly unique to those meeting criteria for ASD at differing levels of ToM and verbal ability, and may thus provide a valuable indicator of deficits and treatment response within this population based on individual profile of cognitive abilities.

This study also suggests that supposedly established predictors of pretend (verbal ability and ToM) do not appear to relate to pretending when a more rigorous measure is applied, indicating that these relations in previous studies may be due to experimenter effects (as suggested by Lillard et al., 2013). On the other hand, that ToM related to a theoretically-similar subtype of pretend (APP) suggests that pretending may be a more complex, multifaceted construct, and that consideration of subtypes may provide a better window into play.

Additionally, this study suggests that interest in toys might be an important predictor of certain subtypes of pretend, specifically OS, that until now have gone unexamined. Furthermore, the finding that interest was a strong predictor of OS among participants, and that this relation differed based on number of ASD symptoms, suggests that interest might prove to be a predictor of pretend among individuals with ASD.

Finally, that interest in toys predicts OS suggests possible implications regarding how certain types of pretend might develop among children. For instance, interest in an object might lead to holistic exploration of that object, which in turn may result in a child exploring (and pretending) what else that object can be (OS). Findings regarding the subtypes of pretend provide deeper insight into the factors that influence the development of the ability to pretend. By gaining a better understanding of what factors contribute to the development of pretend among both TD children and ASD populations, we are better able to understand why some individuals (such as those with ASD) might have difficulty with certain types of pretend and how pretend might develop differently among these individuals.

## Author Contributions

EFK, AL, and ML designed and conducted data collection; EK, EFK, and ML performed data analysis; all authors wrote the manuscript and approved the final version of the manuscript for submission.

## Conflict of Interest Statement

The authors declare that the research was conducted in the absence of any commercial or financial relationships that could be construed as a potential conflict of interest.
